# Low Birth Weight at Term and Its Determinants in a Tertiary Hospital of Nepal: A Case-Control Study

**DOI:** 10.1371/journal.pone.0123962

**Published:** 2015-04-08

**Authors:** Sudesh Raj Sharma, Smith Giri, Utsav Timalsina, Sanjiv Sudarshan Bhandari, Bikash Basyal, Kusum Wagle, Laxman Shrestha

**Affiliations:** 1 Maharajgunj Medical Campus, Institute of Medicine, Kathmandu, Nepal; 2 The University of Tennessee Health Science Center, Memphis, United States of America; 3 Om Health Campus, Kathmandu, Nepal; Hôpital Robert Debré, FRANCE

## Abstract

Birth weight of a child is an important indicator of its vulnerability for childhood illness and chances of survival. A large number of infant deaths can be averted by appropriate management of low birth weight babies and prevention of factors associated with low birth weight. The prevalence of low birth weight babies in Nepal is estimated to be about 12-32%.Our study aimed at identifying major determinants of low birth weight among term babies in Nepal. A hospital-based retrospective case control study was conducted in maternity ward of Tribhuvan University Teaching Hospital from February to July 2011. A total of 155 LBW babies and 310 controls were included in the study. Mothers admitted to maternity ward during the study period were interviewed, medical records were assessed and anthropometric measurements were done. Risk factors, broadly classified into proximal and distal factors, were assessed for any association with birth of low-birth weight babies. Regression analysis revealed that a history of premature delivery (adjusted odds ratio; aOR5.24, CI 1.05-26.28), hard physical work during pregnancy (aOR1.48, CI 0.97-2.26), younger age of mother (aOR1.98, CI 1.15-3.41), mothers with haemoglobin level less than 11gm/dl (aOR0.51, CI0.24-1.07) and lack of consumption of nutritious food during pregnancy (aOR1.99, CI 1.28-3.10) were significantly associated with the birth of LBW babies. These factors should be addressed with appropriate measures so as to decrease the prevalence of low birth weight among term babies in Nepal.

## Introduction

Birth weight is an important determinant of an infant’s survival and future development [1]. Low birth weight (LBW) is defined as weight less than 2500 grams at birth regardless of gestational age. LBW puts a newborn at increased risk of death and illness [2–6] and limits their growth potential in the adulthood [7–9]. Globally, LBW contributes to 40–60% of newborn mortality [10]. LBW can be caused by preterm birth or by intrauterine growth restriction. The latter group is also referred to as small for gestational age (SGA) babies. In developing countries from Asia, LBW is largely attributed to intrauterine growth retardation as compared to prematurity in developed and African countries [11,12]. In Nepal, studies have shown that the prevalence of LBW ranges from 11.9% to 39.6% [13–15]. A multi-hospital based study in Nepal estimated the overall prevalence of LBW to be 27% out of which only 30% were preterm [16].

Prior studies from the Indian subcontinent have identified several socio-economic factors in addition to gestational age and maternal health as risk factors for SGA babies in this region [16–18]. However, studies from Nepal have inadequately addressed the socio-economic factors associated with the birth of LBW babies [19–23]. Many of these studies are based on retrospective chart review that often lack information such as physical work during pregnancy [19,21–23], nutritious food intake [19,21–23] and nutritional supplementation [22], illness during pregnancy [21,22] and household income [19]. Also, these studies have studied LBW babies in general and not separately analyzed the SGA group, even though previous literature has suggested different risk factors for pre-term LBW and SGA babies [24]. To fill the information gap about factors associated with LBW in term babies, we designed the present study in an attempt to identify proximate and distal factors associated with LBW in Nepal.

## Methods

A retrospective case-control study design was used. The study was conducted in Tribhuvan University Teaching Hospital (TUTH), one of the largest tertiary hospitals in Kathmandu, Nepal. Cases were identified as newborn babies with birth weight less than 2500 grams whereas controls were newborn babies with a birth weight of more than or equal to 2500 grams. Our study population included singleton newborn babies at term without any congenital diseases. The study was limited to SGA babies at term only. Unlike in developed countries, majority of LBW babies in the developing countries are small for gestational age due to intrauterine growth retardation, which has been associated with a variety of socio-economic and maternal risk factors [11,12,16].

A total of 155 eligible consecutive cases of LBW babies at term were admitted to TUTH between February 2011 and July 2011. Age and sex matching with two controls was done on the same day when a case was found. Whenever there were more than 2 eligible controls for a case, controls were randomly selected. A total of 310 controls were selected. The non-selected controls were included for other cases on the same day if it met the inclusion criteria (i.e. age and sex matched). Due to high volume of deliveries, controls for a case were found on the same day. The number of cases and controls was calculated by taking power at 80%, odds ratio of 1.8, two sided significant level at 0.05 and proportion of controls with exposure as 0.25 using Epi Info (Version 3.5.2).

The data was collected by interviewing the mothers, observing medical records of the newborn and measuring their anthropometry. Birth weight was measured by the study investigators using a digital non-hanging type salter scale and rounded to the nearest 10 grams. The instrument was calibrated each time before use with a standard 1 kilogram weight. Data were collected by medical and public health students who were properly oriented on data collection techniques by the principle investigator. A structured questionnaire was adapted from previous study by WHO in South-East Asia region and other studies conducted in this region. The tools were pretested during the month of January in Tribhuvan University Teaching Hospital itself in five percent of the sample. The ratio of cases and controls in pretesting was 1:1. Necessary amendments were made and measuring tools were checked properly prior to data collection. Thus collected data was reviewed, edited and re-coded. The study was ethically approved by Institutional Ethical Review Board of Maharajgunj Medical Campus, Institute of Medicine. Respondents were clearly explained about the objective and voluntary nature of the study. Written consent was taken from the respondents and their guardians prior to the interview. Participants were explained about their right to refuse participation and withdraw from the study.

### Explanatory variables

The explanatory variables were divided into proximate and distal factors. The proximate factors included history of chronic medical illness, history of abortion, history of premature delivery, hard physical work during pregnancy, illness during current pregnancy, age of mother, parity, height of mother, haemoglobin level, the practices of taking iron tablets, consuming nutritious food (beans, greens or meat) daily, drinking alcohol during pregnancy and antenatal care (ANC) visits during last pregnancy. Chronic medical illness was defined as a pre-existing medical illness of the mother that was documented in the medical record of TUTH with an onset prior to the current pregnancy. Regular physical work during pregnancy was identified as moderate or hard. Hard physical work comprised of three or more of the following listed works: daily household chores, fetching water with large buckets, lifting heavy loads, chopping woods, cutting grass for cattle feeding and washing clothes/utensils for long. These works have been accounted under hard physical activity in a study among rural women in India [25]. On the other hand, illness during current pregnancy was defined as a medical condition that developed during current pregnancy for which medical attention or treatment was sought. This included pregnancy related medical illnesses and complications based on the International Classification of Diseases (ICD), 10th revision [26]. The age of mother was defined as the current completed age in years. Age of mothers was categorized into less than 20 years, 20 to 30 years and more than 30 years based on a prior study [27]. Parity was categorized into primiparous (mothers with a single living child) and multiparous (mothers with more than one living children). Similarly, mothers were placed into two groups on the basis of their height; less than146 cm and 146 cm or more.

Participants were categorized into two groups based on haemoglobin level; one with haemoglobin level less than 11gm/dL and the other with haemoglobin level 11gm/dL or more based on a prior study [28]. The iron intake frequency of mothers was classified as intake for a period of three months or more and less than three months. Number of ANC visit was classified on the basis of minimum recommended visits i.e. having four or more visits and less than four visits.

The distal factors included education and occupation status of the parents, ethnicity, place of residence, type of family and income of the family. Parental educational status was grouped as illiterate, primary, lower-secondary, secondary, intermediate, bachelors and above. They were further categorized into primary level or less and secondary or above. Occupations of the parents were categorized into five groups; agriculture, service, business, household works and others. These occupations are grouped into either labor intensive or non-labor intensive groups. Labour intensive occupation included more physical works under lower positions and with comparatively less wages. Ethnicity of the mother was first categorized into six major ethnic groups (Brahmin/Chhetri, advantaged Janajati, disadvantaged Janajati, Other excluded Terai group, Religious minorities and Dalit) as per the classification of the Health Management Information System of Ministry of Health/Nepal [29]. In the second step, they were grouped into two as advantaged and disadvantaged group. Brahmin, Chhetri, and advantaged Janajati were grouped into advantaged ethnic group whereas other excluded Terai group, religious minorities, disadvantaged Janajati and Dalit were grouped into disadvantaged ethnic group. This grouping was based on socio-economic and educational difference among these ethnic groups [30]. Place of residence was classified as rural and urban. Type of family was categorized into three groups; nuclear, joint and extended. A nuclear family was defined as mother, father and children living together, whereas joint family was defined as all first-degree blood relatives of many generations living under the same roof. An extended family, on the other hand, was defined as a family extending beyond the immediate family of parents and their children. The yearly income of the family was hypothetically divided into two levels as more than NRs 240,000 and NRs 240,000 or less. The socio-demographic information was recorded based on face-to-face interview with mothers.

### Statistical methods

The data was entered in Epi info (Version 3.5.2)and analyzed in Statistical Package for Social Sciences (Version 17). Bivariate analysis was first conducted with chi-squared test to assess the differences of the various proximal and distal factors between the cases versus the controls (see Table 1 and Table 2). All variables with p-value less than 0.1 (90% level of significance) in bivariate analysis were considered for multivariate logistic regression analysis. Backward stepwise logistic regression method was used to enter the predictive factors in logistic regression model. The level of significance for regression analysis was set at 95%. All p values were two sided.

**Table 1:** Description of proximal factors and their association with low birth weight.

Proximal factors	No. (%) of Cases (n = 155)	No. (%) of Controls (n = 310)	p-value
**History of chronic medical Illness to mother**			
No	146 (94.2)	298 (96.1)	Ref.
Yes	9 (5.8)	12 (3.9)	0.343
**History of abortion**			
No	131 (84.5)	274 (88.4)	Ref.
Yes	24 (15.5)	36 (11.6)	0.24
**History of premature delivery**			
No	147 (94.8)	308 (99.4)	Ref.
Yes	8 (5.2)	2 (0.6)	0.002
**Hard physical work during pregnancy**			
No	84 (54.2)	207 (66.8)	Ref.
Yes	71 (45.8)	103 (33.2)	0.008
**Illness during current pregnancy**			
No	140 (90.3)	292 (94.2)	Ref.
Yes	15 (9.7)	18 (5.8)	0.125
**Current age category**			
Less than 20 years	31 (20)	38 (12.3)	Ref.
Age between 20–29 years	108 (69.7)	234 (75.5)	
More than 30 years	16 (10.3)	38 (12.3)	0.083
**Parity category**			
Multiparous	31 (20.0)	73 (23.5)	Ref.
Primiparous	124 (80.0)	237 (76.5)	0.387
**Height of mother**			
Height 146cm or more	139 (89.7)	292 (94.2)	Ref.
Height less than 146cm	16 (10.3)	18 (5.8)	0.078
**Haemoglobin level**			
Hb level 11 or more	10 (6.5)	40 (12.9)	Ref.
Hb level less than 11	145 (93.5)	270(87.1)	0.039
**Iron intake frequency 3 months**			
Iron intake 3 months or more	143 (92.3)	292 (94.2)	Ref.
Iron intake less than 3 months	12 (7.7)	18 (5.8)	0.423
**Ate beans, greens or meat daily during pregnancy**			
Yes	91 (58.7)	236 (76.1)	Ref.
No	64 (41.3)	74 (23.9)	0.000
**Alcohol intake during pregnancy**			
No	154 (99.4)	306 (98.7)	Ref.
Yes	1 (0.6)	4 (1.3)	0.512
**ANC visits**			
4 or more times	150 (96.8)	290 (93.5)	Ref.
Less than 4 times	5 (3.2)	20 (6.5)	0.146

**Table 2:** Description of distal factors and their association with low birth weight.

Distal factors	No (%) of Cases (n = 155)	No (%) of Controls (n = 310)	p-value
**Education of mother**			
Secondary level and above	134 (86.5)	251 (81.0)	Ref.
Primary level or less	21 (13.5)	59 (19.0)	0.140
**Education of father**			
Secondary level and above	145 (93.5)	284 (91.6)	Ref.
Primary level or less	10 (6.5)	26 (8.4)	0.462
**Occupation of mother**			
Non-labour intensive	40 (25.8)	77 (24.8)	Ref.
Labour intensive	115 (74.2)	233 (75.2)	0.821
**Occupation of father**			
Non-labour intensive	154 (99.4)	301 (97.1)	Ref.
Labour intensive	1 (0.6)	9 (2.9)	0.114
**Ethnicity**			
Brahmin/Chhetri (Advantaged)	138 (89.0)	251 (81.0)	Ref.
Dalit/Janjati (Disadvantaged)	17 (11.0)	59 (19.0)	0.027
**Place of residence**			
Urban	151 (97.4)	297 (95.8)	Ref.
Rural	4 (2.6)	13 (4.2)	0.382
**Type of family**			
Nuclear	99 (63.9)	222 (71.6)	Ref.
Joint/Extended	56 (36.1)	88 (28.4)	0.089
**Yearly income**			
More than NRs 240000	66 (42.6)	114 (36.8)	Ref.
NRs 240000 or less	89 (57.4)	196 (63.2)	0.226

## Results

A total of 155 cases and 310 controls were studied. Tables 1 and 2 show the distribution of cases and controls as per proximate and distal factors respectively.

Chronic medical illness was present only in a small percentage of the study population. Majority of the mothers had no prior history of abortion or premature delivery. Engaging in hard physical work during pregnancy was fairly common (46% and 33% among cases and controls respectively). Majority of mothers belonged to age 20 to 29 years (69% among cases and 75% among controls), had a height of 146 cm or more (89% among cases and 94% among controls). More than 90% of both groups were taking iron supplements since at least 3 months. Majority of mothers (93% cases and 87% controls) had their haemoglobin level less than 11gm/dl. Alcohol intake during pregnancy was fairly low (0.6% and 1.3% respectively) and majority of mothers had an ANC visit of at least 4 times (96.8% and 93.5% respectively) as in Table 1. Among the distal factors (Table 2), majority of mothers had at least secondary level of education (86% and 81% respectively), belonged to an advantaged ethnicity (89% and 81% respectively) and urban residence (97% and 96% respectively). Similarly 57.4% of the cases and 63.2% of the controls had a yearly income of less than NRs 240,000 or less.

### Determinants of LBW

Results of the bivariate (Tables 1 and 2) and the multivariate analysis (Table 3) to study the association between different risk factors and the occurrence of LBW have been summarized on Tables 1–3. In bivariate analysis, history of premature delivery, hard physical work done during pregnancy, current age of mother, height of mother, haemoglobin level, consuming nutritious food during pregnancy, ethnicity and family type were significantly associated with LBW at 90% level of significance (p-value 0.1). These variables were then subjected to the multivariable analysis using backward stepwise logistic regression method (Table 3). History of premature delivery (aOR5.24, CI 1.05–26.28, hard physical work during pregnancy (aOR1.48, CI 0.97–2.26), younger age of mother (aOR1.98, CI 1.15–3.41), mothers with haemoglobin level less than 11gm/dl (aOR0.51, CI 0.24–1.07) and consumption of nutritious food during pregnancy (aOR1.99, CI 1.28–3.10) were the determinants of LBW, found significantly associated in multivariate analysis.

**Table 3:** Results from the stepwise logistic regression model showing Independent risk factors of low birth weight.

Independent Variables	Full Model		Reduced Model	
	Adjusted odds ratio (95% CI)	P value	Adjusted odds ratio (95% CI)	P value
**History of premature delivery**				
No	1	Ref.	1	Ref.
Yes	4.65 (0.94–22.98)	0.06	5.24 (1.05–26.28)	0.04
**Hard physical work**				
No	1	Ref.	1	Ref.
Yes	1.48 (0.96–2.26)	0.07	1.48 (0.97–2.26)	0.07
**Age group**				
20–30	1	Ref.	1	Ref.
Less than 20	2.15 (1.23–3.76)	0.01	1.98 (1.15–3.41)	0.01
More than 30	0.91 (0.46–1.78)	0.78	0.84 (0.44–1.62)	0.61
**Haemoglobin level**				
11 g/dl or more	1	Ref.	1	Ref.
Less than 11 g/dl	0.52 (0.25–1.09)	0.08	0.51 (0.24–1.07)	0.08
**Ate meat, bean and green vegetables regularly**				
Yes	1	Ref.	1	Ref.
No	1.87 (1.19–2.94)	0.01	1.99 (1.28–3.10)	0.002
**Height of Mother**				
> 146 cm	1	Ref.	-	-
< 146 cm	1.65 (0.79–3.48)	0.18	-	-
**Ethnicity**				
Brahmin/Chhetri (Advantaged)	1	Ref.	-	-
Dalit/Janjati (Disadvantaged)	0.71 (0.45–1.12)	0.14	-	-
**Type of family**				
Nuclear	1	Ref.	-	-
Joint/Extended	0.79 (0.51–1.23)	0.29	-	-

## Discussion

Based on the findings of the present study, history of premature delivery, hard physical work during pregnancy, earlier age at pregnancy, lack of consumption of nutritious food during pregnancy and maternal anemia were the independent risk factors for the birth of SGA babies in Nepal.

Our study revealed that a history of premature delivery was significantly associated with LBW in babies. A study by Singh et al in Ahemdabad, India also showed similar findings [31]. A highly significant association of history of premature delivery with LBW in this study indicated that mothers with history of premature delivery may need special care during pregnancy in terms of diet and health check-up.

Hard physical work during pregnancy was found to be significantly associated with LBW in this study. A greater proportion of LBW babies were born to mothers who had done hard physical work during pregnancy. A study from Lithuania depicted similar findings that hard manual work by mothers attributed to greater percent of LBW cases [32]. Agarwal et al also showed similar relationship between physical work of mother and LBW [33]. Lifting heavy loads during pregnancy has been shown to be one of the risk factors for low birth weight [34]. One mechanism suggested involves placental hypoxia leading to low birth weight babies, particularly among undernourished women [25]. Frequency and duration of rest taken by pregnant women plays a significant role in determining birth weight [35].

Many studies have shown age of the mother to be significantly associated with the birth of LBW babies [33,36–41]. Previous studies have shown that younger mothers are more likely to have LBW babies [32,42]. The current study showed that young mothers (less than 20 years of age) were nearly two times more likely to deliver LBW babies compared to older mothers. Efforts towards preventing early marriage would contribute significantly in reducing the prevalence of low birth weight.

The current study well found mother’s level of haemoglobin to be associated with LBW. Lower concentration of maternal haemoglobin is one of the risk factors for LBW among children [43,44]. Maternal anaemia further limits maternal oxygen uptake, decrease oxygen delivery to fetus [45] and consequently leads to fetal growth restriction [46,47]. Various studies have previously depicted that anemic mothers with haemoglobin level less than 11gm/dl have higher chances of giving birth to LBW babies [48–50].

The study also found that consumption of nutritious diet during pregnancy affects the birth weight, a well-established fact [51–54]. Poor maternal food intake leads to fetal under-nutrition and deficiency of several micronutrients which are necessary for the growth and development of fetus [55,56]. This finding implicates that the prevalence of low birth weight can be reduced by promoting consumption of meat, beans and green vegetables by pregnant mothers and mitigating food related taboos.

This study however had some notable limitations. The findings might be influenced by purposive selection of study area, study design bias and social-desirability bias. Use of hospital records for background information may have introduced some bias. The study was based on a single tertiary institution limiting its generalizability. In a country with proportion of home deliveries about 65% [57], a community based study may provide more clear picture on the determinants of low birth weight. Also, we did not evaluate other potential risk factors for low birth weight including micronutrient deficiencies among mothers, urinary tract or genital infections, cigarette smoking, other toxic exposures, and the quality of antenatal care received by the mothers, which may have affected our results [58].

## Conclusions

History of premature delivery, hard physical work during pregnancy, younger age at pregnancy, mother’s lower haemoglobin level and lack of nutritious diet consumption during pregnancy were the major determinants of low birth weight among term babies in Nepal. Public health programs should focus on raising awareness on avoiding early marriage and pregnancy of females. Moreover, it should provide emphasis on adequate rest and nutrition during pregnancy in order to decrease the prevalence of low birth weight. Provision of a more intensive ANC to mothers with a history of premature deliveries can be another important strategy to prevent low birth weight babies. The role of family members is important especially in fulfilling the nutritional and health care needs of the pregnant mothers along with supporting her to take adequate rest in the cultural context of Nepal.
